# Can whitening toothpastes maintain the optical stability of enamel over time?

**DOI:** 10.1590/1678-7757-2016-0460

**Published:** 2018-01-16

**Authors:** Eduardo Moreira da Silva, Juliana Nunes da Silva Meirelles Dória Maia, Carine Gnatiuk Mitraud, Juliana do Espírito Santo Russo, Laiza Tatiana Poskus, José Guilherme Antunes Guimarães

**Affiliations:** 1 Universidade Federal Fluminense, Faculdade de Odontologia, Laboratório Analítico de Biomateriais Restauradores, Niterói, RJ, Brasil

**Keywords:** Dental enamel, Toothbrushing, Toothpastes, Surface properties, Color

## Abstract

Besides the effects on the health of individuals, cigarette smoking can also interfere with the appearance of their teeth.

**Objective:**

To evaluate the effect of cigarette smoking-toothbrushing-cycling (CSTC) with whitening toothpastes on the roughness and optical behavior of bovine enamel for eight weeks.

**Material and Methods:**

Thirty bovine dentin/enamel discs, 8.0 mm in diameter and 2.0 mm thick, were randomly divided into three groups according to the toothpastes: whitening (Colgate Luminous White - CW and Oral B 3D White - OW), and a non-whitening (Colgate - C). The roughness, color (CIE L*a*b* system), translucency and gloss were measured before and after the specimens were submitted to CSTC. The topography of the specimens was analyzed by scanning electron microscopy. During the first week, the specimens were daily subjected to the consumption of 20 cigarettes and brushed (40 strokes/100 g) with the toothpastes' slurries. Thereafter, the CSTC was weekly applied in an accumulated model (140 cigarettes/280 strokes) for seven weeks. The data were submitted to two-way ANOVA, Tukey's HSD test, and paired-t test (α=0.05).

**Results:**

The three toothpastes produced significant changes in roughness, color, translucency and gloss (p<0.05). After eight weeks, the roughness and the gloss produced by the three toothpastes were similar (p>0.05), while OW produced the lowest color change and the translucency of C was lower than that of CW (p<0.05). The three toothpastes produced a significant decrease in L* values and a significant increase in a* values after eight weeks (p<0.05). No significant difference in the b* coordinate was found for OW (p=0.13) There were topographic changes in the enamel surfaces.

**Conclusions:**

The whitening toothpastes increased the roughness, changed the topography and were not able to maintain the optical stability of enamel exposed over eight weeks.

## Introduction

Among others, cigarette smoking is one of the most deleterious habits that cause devastating effects, such as cancer, emphysema, bronchitis, and coronary disease, on individuals^31^. Unfortunately, according to the World Health Organization (WHO), more than one billion people use tobacco around the world and six million die *per* year due to this habit^32^. This picture is a matter of concern.

In addition to a great variety of toxic chemicals, e.g., naphthalene, hexane, formaldehyde, carbon monoxide, arsenic, ammonia, and toluene, tobacco also contains staining substances, such as tar and coffee, that may cause extrinsic discoloration of teeth^3^, and restorative biomaterials^1^. From the point of view of Dentistry, this aspect also represents an aesthetic concern. Specifically in terms of tooth discoloration, it has been shown that bleaching techniques that use hydrogen peroxide and other substances are efficient to remove intrinsic and extrinsic staining produced by different sources^7^ ^,^ ^23^. Clinically, the patient should avoid the use of staining substances during the whitening protocols that use these chemical stain removers^10^. However, this is not an easy task for smokers. Thus, the previously named whitening toothpastes seem to be an alternative path to these patients.

Besides the basic ingredients used in traditional products, e.g., surfactants, thickening agents, flavor, and fluorides, whitening toothpastes also contain higher amounts of abrasives that are capable of removing or preventing the deposition of extrinsic stains on the tooth's surface^11^. The most common abrasives used in today's whitening toothpastes include hydrated silica, calcium carbonate, dicalcium phosphate dihydrate, sodium bicarbonate, perlite, and alumina^8^. During the toothbrushing, a three-phase system formed by the tooth surface, the toothbrush bristles, and the abrasives between these are responsible for stain removing^14^. However, depending on the hardness, shape and the size of abrasives, toothbrushing may also wear the tooth surface and cause changes in color and roughness^9^.

Although the results presented in the scientific literature have added important aspects to the comprehension of the action of whitening toothpastes on the enamel surfaces, there is still a lack of sound information on their action over enamel submitted to cigarette smoking^3^. Therefore, the purpose of our study was to conduct an *in vitro* investigation about the influence of a cigarette smoking-toothbrushing- cycling (CSTC) by using whitening toothpastes on the roughness and the optical stability (color, translucency and gloss) of bovine enamel over a period of eight weeks. The null hypothesis tested was that no toothpaste would influence the roughness and the optical stability of bovine enamel after eight weeks of exposure to cigarette smoking-toothbrushing-cycling.

## Material and methods

**Figure 1:**
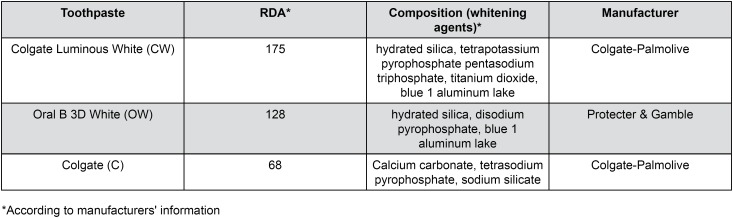
Composition of the toothpastes analyzed in this study

Thirty bovine incisors selected according to similar color and maintained in a 0.2% thymol solution at 4°C for one week were used in this study. Before the specimens' preparation, the teeth were examined under a stereomicroscope at 10x magnification (SZ40, Olympus, Tokyo, Japan) to identify the presence of any defects that could interfere with the obtained results. The roots were separated from the crowns and the teeth were sectioned through the pulp chamber using a low speed water-cooled diamond saw (Isomet 1000, Buehler, Lake Bluff, IL, USA) to obtain enamel/dentin slices from their labial surfaces. The enamel and dentin surfaces of each slice were ground flat with 1200-, 2500-, and 4000-grit SiC papers (DPU-10, Struers, Copenhagen, Denmark), which was controlled with a digital caliper (MPI/E-101, Mitutoyo, Tokyo, Japan), until reaching a thickness of 2.0±0.1 mm (1.0 mm of dentin and 1.0 mm of enamel). Afterwards, disc-shaped enamel/dentin specimens with 8.0 mm in diameter were prepared from each slice by using a diamond bur (#3097, KG Sorensen, Cotia, SP, Brazil) in a highspeed hand piece fixed in a special sample-aligning device. The specimens were randomly divided into three groups of ten specimens according to the three toothpastes analyzed (Figure 1) and kept in artificial saliva at 37°C before taking all measurements.

### Baseline measurements

#### Roughness

All specimens had their surface roughness evaluated by using a surface roughness tester (Surftest SJ 201, Mitutoyo, Tokyo, Japan). Four traces of roughness spaced at 45°, with a 0.8 mm cutoff and a speed of 0.1 mm/s, were recorded for each specimen, and the average surface roughness *(R_a_* - μm) was determined. The *R_a_* parameter was obtained using the following formula:

$$R_{a}=\frac{1}{L}\int_{0}^{L}\left|f(x)\right|dx$$

where L is the length of the section and *f*(x) is the displacement function.

### Color and translucency

The color was measured according to the CIE L*a*b* system by using a spectrophotometer (model CM2600d, Konica Minolta Sensing Inc., Osaka, Japan). A D65 illuminant, under 100% UV energy and specular reflection included (SCI), was used with a 45° entrance angle and 0° observation angle geometry. We carried out the measurements using a small area view (SAV). Before each measurement session, the spectrophotometer was calibrated by using the white calibration standard supplied by the manufacturer. In order to guarantee the consistency of consecutive and repeated measurements of CIE L*a*b* parameters, they were carried out over white and black spectrophotometry ceramic standards (Konica Minolta Sensing Inc., Osaka, Japan) that were precisely attached to the base unit of the spectrophotometer by using a customized jig with a central hole where the specimens were positioned. This procedure allowed the color to be consistently measured in the central area and at the same position for all the specimens. The L*, a* and b* values of each specimen were separately measured in triplicate against the white and the black backgrounds.

### Gloss

Gloss was measured by using a small-area glossmeter (ZGM 1110, Zehntner testing instruments, Sissach, Switzerland), with a square measurement area of 2 mm x 2 mm and 60° geometry. A custom-made jig was used to place the specimen over the aperture of the glossmeter at the same position at each time of measurement. The gloss, expressed in gloss units (GU), was measured in triplicate for each specimen.

### Cigarette Smoking-Toothbrushing-Cycling - CSTC

**Figure 2:**
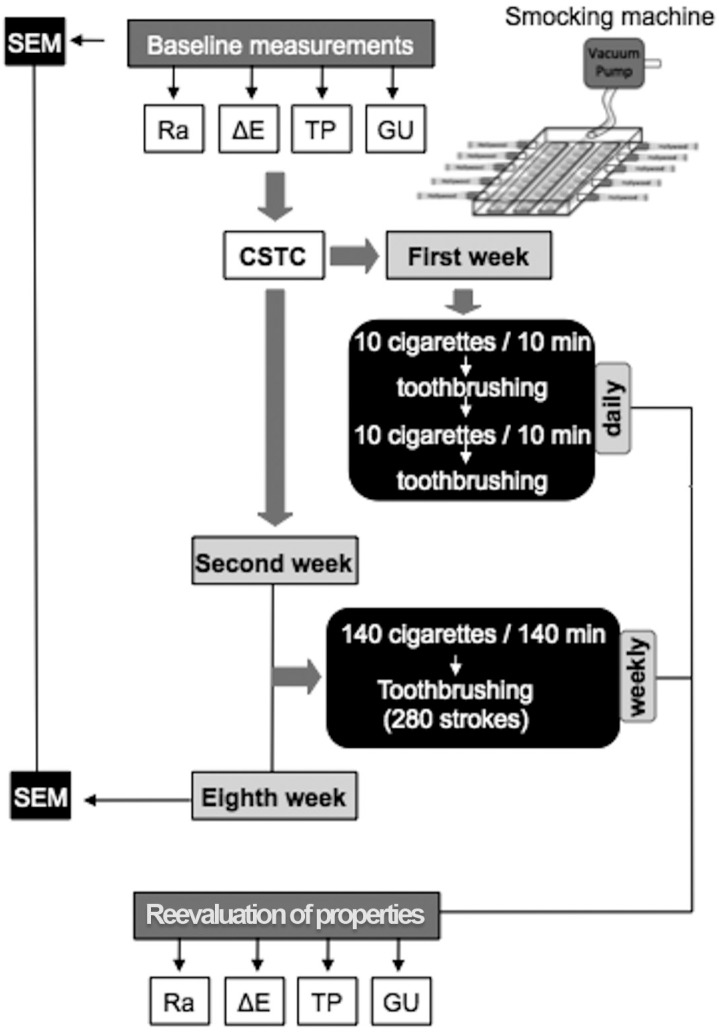
Flowchart of the study

After the baseline measurements, the specimens were submitted to CSTC (Figure 2). During the first week, the specimens were daily exposed to 20 cigarettes (Hollywood, Souza Cruz, Cachoeirinha, RS, Brazil) by using a cigarette smoking machine. This consisted of a hermetically closed acrylic box with five holes on each side to fit the cigarettes and internal supports that allowed the specimens to be positioned with the enamel surfaces facing up. The smoking machine was connected to a vacuum pump by a silicone tube that caused a negative pressure enough to consume and aspirate the smoke released by the cigarettes. The specimens were exposed to smoke produced simultaneously by 10 cigarettes for 10 min. Then, each specimen was brushed [20 strokes/100 g + toothpaste slurry in a proportion of 1:2 by weight (18 g of each toothpaste and 36 mL of artificial saliva)] in a brushing machine (MEV2, Odeme Biotechnology, Joaçaba, SC, Brazil). After that, the specimens were again exposed to 10 cigarettes and brushed using the same parameters. Between the daily cycles, the specimens were stored in artificial saliva (KCl, NaCl, MgCl, CaCl, Nipagin, CNC, Sorbitol, and deionized water - pH = 7) at 37°C. The CSTC was repeated every day for seven days. After the first week, the specimens were maintained in artificial saliva at 37°C and resubmitted to CSTC once a week for a period of eight weeks in a cumulative model (7×20 cigarettes + 7×40 strokes).

### Reevaluation of properties

All the properties (roughness, color, translucency, and gloss) were reevaluated after each CSTC (daily during the first week and weekly from the second to the eighth week). The color change (*ΔΕ*) for each specimen was calculated from the mean ΔL*, Δa*, and Δb* values, which were obtained against the white background, by using the following formula:

$$\text{Δ}E={\left[{\left(\text{Δ}L^{*}\right)}^{2}+{\left(\text{Δ}a^{*}\right)}^{2}+{\left(\text{Δ}b^{*}\right)}^{2}\right]}^{1/2}$$

where ΔL*, Δa*, Δb* are the differences in L*, a* and b* coordinates obtained before and after each subsequent CSTC.

The L*, a* and b* coordinates obtained on the first and second day of evaluation were used to calculate the *ΔΕ* at baseline.

The translucency parameter (TP) for each specimen after each day (when measured daily) and each week (when measured weekly) was calculated using the following formula:

$$TP={\left[{\left(L_{B}-L_{W}\right)}^{2}+{\left(a_{B}^{*}-a_{W}^{*}\right)}^{2}+(b_{B}^{*}-b_{W}^{*})\right]}^{1/2}$$

where the subscript B and W letters represent the measurements against the black and white backgrounds, respectively, in each subsequent CSTC.

### Topographic analysis

Two specimens from each group, randomly selected, were analyzed by scanning electron microscope (SEM) at baseline and after the eighth week. The specimens were mounted in a charge reduction sample holder and observed under SEM (Phenom ProX, Phenom World, Eindhoven, Netherlands) operating in backscattered mode in a low vacuum environment. The SEM images were taken by employing 15 Kv, at a magnification of x2500.

### Statistical analysis

We analyzed the obtained data using Statgraphics Centurion XVI software (STATPOINT Technologies, Inc., Warrenton, VA, USA). The normal distribution of errors and the homogeneity of variances were preliminarily checked by Shapiro-Wilk's and Levene's tests. Based on these analyses, roughness, color, translucency and gloss were separately analyzed by two-way ANOVA repeated measures and Tukey's HSD *post hoc* test. We used paired-t test to analyze the differences in L*, a*, and b* coordinates at baseline and after eight weeks of CSTC. All analyses were performed at a significance level of α = 0.05.

## Results

The mean values of roughness, *ΔΕ, TP,* and gloss at baseline and after eight weeks of CSTC are presented in Table 1. The three toothpastes produced significant changes in roughness, *ΔΕ*, *TP,* and gloss after eight weeks of CSTC (p<0.05). For roughness, the values after eight weeks were statistically similar (p>0.05). The *ΔΕ* produced by OW after eight weeks was lower than those produced by C and CW (p<0.05), which were not different from each other (p>0.05). After eight weeks, C produced the lower alteration in enamel translucency (p<0.05), but with no difference from that produced by OW. After eight weeks of CSTC, the gloss produced by the three toothpastes was similar (p>0.05).

**Table 1:** Mean values (±SD) of roughness (μm), ΔΕ, TP and Gloss at baseline and after 8 weeks of cigarette-smoking-toothbrushing-cycle (CSTC)

C		CW		OW	
Baseline	8 weeks	Baseline	8 weeks	Baseline	8 weeks
Roughness					
0.07^a^ (0.02)	0.12^b^ (0.02)	0.06^a^ (0.01)	0.14^b^ (0.03)	0.08^a^ (0.01)	0.13^b^ (0.02)
ΔΕ					
3.2^a^ (0.3)	18.1^b^ (2.3)	3.9^a^ (0.2)	15.3^b^ (2.0)	4.3^a^ (0.5)	10.3^c^ (1.9)
TP					
9.4^a^ (0.5)	4.9^b^ (1.4)	10.6^a^ (1.0)	7.1^c^ (1.1)	11.4^a^ (1.4)	6.7^b,c^(1.6)
Gloss					
70.8^a^ (10.1)	58.2^b^ (6.9)	72.0^a^ (1.8)	60.9^b^ (4.6)	62.9^a^ (8.0)	52.6^b^ (3.8)

In rows, means followed by the same lowercase letter are similar (Tukey HSD test, p>0.05)

The L*, a*, and b* color coordinates of enamel at baseline and after eight weeks are shown in Table 2. The three toothpaste groups presented a significant decrease in L* values and a significant increase in a* values after eight weeks (p<0.05). Conversely, only C and CW showed a significant increase in b* coordinate after eight weeks (p=0.1323).

**Table 2:** Mean values (±SD) of L*, a* and b* coordinates at baseline and after 8 weeks of cigarette-smoking-toothbrushing-cycle (CSTC)

C		CW		OW	
Baseline	8 weeks	Baseline	8 weeks	Baseline	8 weeks
L*					
85.73^a^ (1.87)	70.91^b^ (7.11)	84.75^a^ (1.44)	72.85^b^ (4.31)	84.62^a^ (1.24)	77.09^b^ (4.15)
a*					
0.99^a^ (0.21)	6.68^b^ (1.39)	0.58^a^ (0.13)	6.73^b^ (1.50)	0.69^a^ (0.18)	5.22^b^ (1.10)
b*					
11.98^a^ (1.34)	20.68^b^ (4.55)	12.82^a^ (2.02)	20.04^b^ (3.87)	13.84^a^ (2.05)	17.68^a^ (2.95)

In rows, for each toothpaste, means followed by the same lowercase letter are similar (Paired-t test, p>0.05)

**Figure 3:**
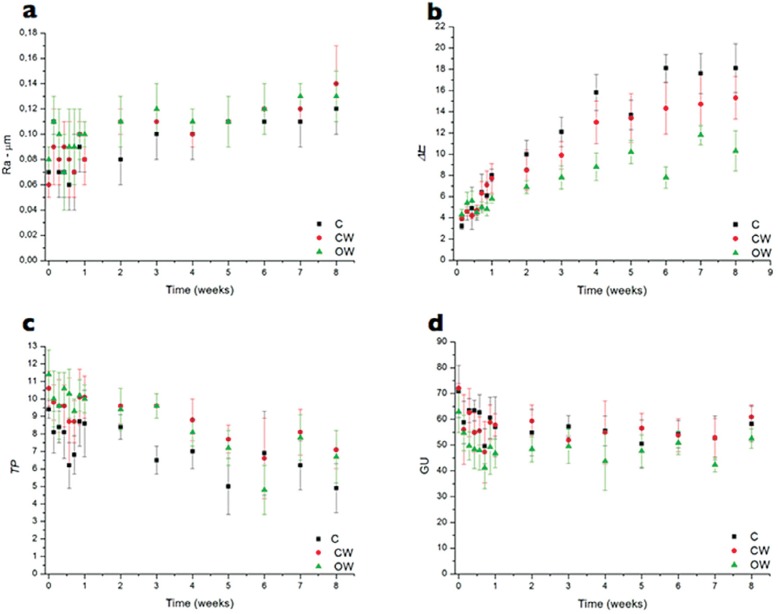
Curves of evolution of properties vs. time for the three toothpastes: roughness (a), color (b), translucency (c) and gloss (d)

Figure 3 shows the evolution of all properties from the baseline to the eighth week of the CSTC. For the three toothpastes, roughness (a) increased uniformly from the second to the eighth week. Color (*ΔΕ*) (b) presented a remarkable change until the fifth week, modifying in a subtle way from the fifth to the eighth week. The translucency (*TP*) (c) decreased in an irregular way, with values ranging up and down. Gloss (d) presented the greatest changes in the first week, and was relatively stable from the second to the eighth week.

**Figure 4:**
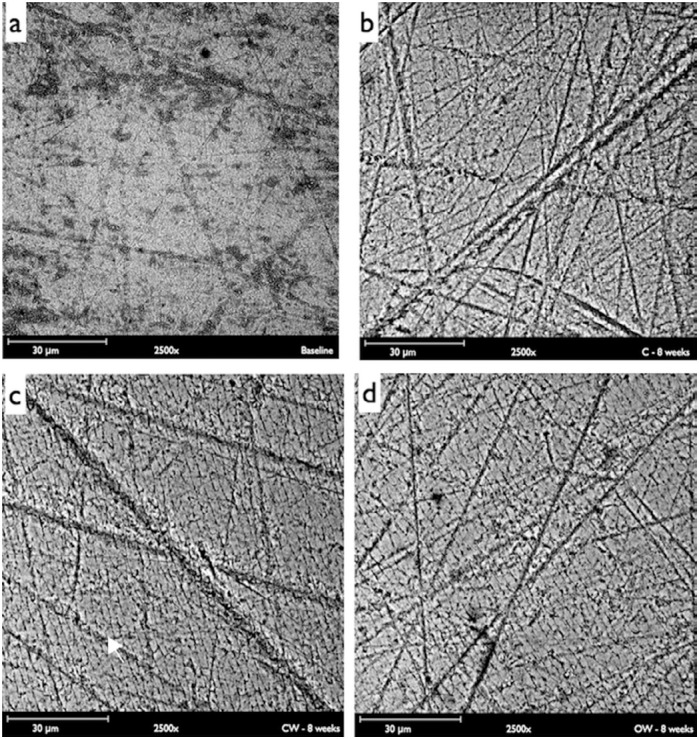
Representative scanning electron microscopy (SEM) micrographs of enamel before (a) and after eight weeks of CSTC: Colgate (b), Colgate Luminous White - CW (c) and Oral B 3D White - OW (d)

Representative SEM micrographs of enamel before (a) and after (b, c, and d) eight weeks of CSTC are depicted in Figure 4. Regardless of the toothpaste used, CSTC produced topographic changes in the enamel surfaces, with the enamel prisms being more evident in the specimens brushed with the whitening toothpastes: CW (c) and OW (d).

## Discussion

In a recent study on the prevalence of smoking and cigarette consumption in 187 countries, Ng, et al.^21^ (2014) showed that, in 2012, 34 countries presented an average of cigarettes consumption per smoker *per* day of less than 10. In 78 countries, this number ranged from 10 to 20 cigarettes, and was greater than 20 in the last 75 countries. This was the basis to use a protocol of the consumption of 20 cigarettes *per* day in this study, analyzing the responses accessed here by using a worst-case scenario. A previous study has shown that moderate (10-20 cigarettes/day) and heavy (>20 cigarettes/day) smokers presented a habit of toothbrushing twice a day^25^. This was the rationale to the strategy of consumption of 10 cigarettes followed by brushing two times in the first week. The idea was to simulate, as closely as possible, a common day in a smoker's life. The number of 40 strokes *per* day to brush the specimens was based on an estimation that a tooth is brushed for 20 s in each daily toothbrushing of 2 min. Thus, considering that a heavy smoker brushes their teeth twice a day^25^, this means that each tooth will be submitted, on average, to 40 strokes daily.

We can note in Table 1 that after eight weeks of CSTC, the three tested toothpastes produced significant alterations on roughness and optical stability [color (*ΔΕ*), translucency and gloss] of bovine enamel. Thus, the null hypothesis of our study was rejected. From the clinical point of view, surface roughness presents crucial importance due to two aspects. First, it exerts a great influence on bacterial adhesion forces^18^, an aspect that may increase the biofilm accumulation and, therefore, produce demineralization of enamel^6^, and periodontal diseases^24^. Second, roughness implies in sulcus formation in the enamel surfaces that can favor the accumulation of oral pigments, e.g., coffee, tea and tobacco, which may interfere with the optical appearance of enamel. This is an aesthetic matter. In our study, we found a significant increase in roughness from the baseline to the eighth week (Table 1), with the baseline and eight week values of roughness nicely agreeing with others previously published^30^. Moreover, Figure 3a shows that this increase in roughness was gradual, characterizing a cumulative effect of toothbrushing on this response. On the other hand, it is noteworthy that the final absolute values of roughness, ranging from 0.12 to 0.14 mm, were below 0.2 mm, which is the crucial number in terms of bacteria accumulation^4^. Thus, these roughness values would not represent a clinical issue from the periodontal and enamel demineralization point of view.

The abrasiveness of toothpastes is strongly influenced by characteristics of the abrasive particles included in their formulations, i.e., hardness, size, shape and size distribution^5^ ^,^ ^11^ ^,^ ^14^. This was considered to analyze the three toothpastes used in this study. Colgate (RDA=68) is a regular toothpaste with less abrasive calcium carbonate particles^19^. Conversely, the whitening toothpastes (CW - RDA = 175 and OW - RDA = 128) have hydrated silica, an intermediate abrasive agent^19^. CW also presents titanium dioxide, which has moderate abrasiveness. Based on these differences, the similarity among the values of roughness of eight weeks produced by the three toothpastes was unexpected. However, some previous studies can support these findings. Hilgenberg and others^9^ (2011) showed similar bovine enamel roughness after 1,600 toothbrushing strokes with a regular calcium carbonate-based (low abrasiveness) and two whitening silica-based toothpastes. Moreover, an analysis of the roughness of human enamel brushed with different toothpastes *in situ* (42 days in the oral environment) by Melo, Manfroi and Spohr^19^ (2014) also showed no differences on enamel roughness produced by a calcium carbonate-based and two hydrated silica-based whitening toothpastes. This last study is noteworthy because, even in the oral environment, a dynamic system and toothpastes with different RDAs produced no differences on enamel roughness.

According to Pascaretti-Grizon, Mabilleau and Chappard^22^ (2013), even having different abrasives, toothpastes can produce similar abrasiveness due to the differences in size and other characteristics of these particles. Thus, we assume that while the three toothpastes analyzed in our study present different abrasives (Table 1), the synergism produced by their size, hardness and or distribution could have influenced the final values of roughness observed here. The features presented in Figure 4 could reinforce this thought, that is, although CW and OW present hydrated silica as abrasive, it seems that the sulcus produced on the enamel surfaces by CW (Figure 4c) were somewhat wider than those produced by OW (Figure 4d), suggesting that the former presents greater abrasive particles.

Different from previous studies^3^ ^,^ ^15^ ^,^ ^28^, the whitening toothpastes in this study were not capable of removing the staining and maintaining the color stability of enamel surfaces after eight weeks (Table 1). Most probably, this result can be explained by the differences between the experimental protocols used in previous and present studies, that is, in those cited studies, the enamel specimens were first exposed only to staining solutions (black tea^15^ or coffee^28^) or to a coffee solution plus cigarette smoking^3^ and then submitted to toothbrushing. Certainly, this staining protocol favored the formation of an extrinsic stained pellicle onto the enamel surfaces, which was easily removed by the subsequent toothbrushing^2^. In this study, the enamel surfaces were alternately submitted to cigarette smoking and toothbrushing during the entire experimental protocol. Thus, it is possible that in each CSTC, the products derived from the cigarette smoke could have impregnated the sulcus produced by toothpaste abrasives (Figure 4) and the dissimilarities between the diameter of the toothbrush filaments and the width of those sulcus could have prevented the abrasive particles from reaching these deeply stained areas^14^.

Another important aspect observed here is that all the values of *ΔΕ* after eight weeks were greater than 3.3, which is the clinically acceptable value for color changing^26^. Moreover, from the data shown in Figure 3b, the progressive *DE* was clearly a cumulative phenomenon, reinforcing that none of the toothpastes was capable of maintaining the color stability over time. Regardless, the curves depicted in Figure 3 show that during the eighty weeks the *ΔΕ* of CW and OW were lower than those of C. Most probably, the optical whitening agents present in CW and OW (Table 1) influenced this behavior. Blue 1 aluminum lake is an optical whitening agent proposed to be deposited onto the tooth surfaces and to create a blue color perception that increases the whitening effect.

The CIE L*a*b* system represents a threedimensional color space that provides a representation for the perception of color stimuli, where the L* axis measures the lightness of the object, ranging from 0 (black) to 100 (white) and the a* and b* axes represent the degree of green-red and blue-yellow color, respectively^12^. In our study, all toothpastes produced a significant decrease in L* values after eight weeks (Table 2). From this finding, we can interpret that the enamel underwent a reduction in lightness, regardless of the toothpaste used. Also, the significant increase in a* and b* values indicates a tendency to discoloration to dark brown and dark yellow and reinforces the fact that no toothpaste was capable of removing the staining produced by the cigarette smoking. Furthermore, the fact that OW did not present a significant increase in b* value and presented a higher numerical L* coordinate after eight weeks can be possibly explained by a greater amount of Blue 1 aluminum lake in its composition.

Translucency can be defined as the relative quantity of light transmission or diffuse reflection from a material surface through a turbid medium. In enamel, this phenomenon is influenced by its complex microstructure (crystals and prisms)^27^, and, among other things, by micromorphological surface modifications^13^. In this field, Ma, et al.^16^ (2009) showed that the translucency of enamel was reduced after 14 days of bleaching with carbamide peroxide and linked this finding to enamel surface alterations produced by the bleaching protocol. This behavior is corroborated by the study of Vieira, Arakaki and Caneppele^29^ (2008). In our study, the three toothpastes produced a progressive decrease in enamel translucency from the first to the eighth week (Figure 3c), reaching significantly lower *TPs* at the end of the experimental protocol (Table 1). Most likely, these results were influenced by the increase in roughness after each CSTC (Figure 3a), which could have increased the diffuse reflectance onto the enamel surfaces, thereby lowering its translucency^13^. This decrease in translucency observed here could have also influenced the results of color evaluation. Tooth color is the result of diffuse reflectance from the inner dentine through the outer translucent enamel^17^. Thus, if the translucency of the enamel was reduced, clearly, less light from the dentine was captured by the spectrophotometer. This thought is supported by the study by Ma, et al.^17^ (2011) who showed that the tooth color change was influenced by the lowering of the translucency of enamel after bleaching.

Although the three toothpastes produced a statistically significant difference on gloss between the first and the eighth week (Table 1), this was the optical property least affected by the CSTC in our study. In fact, the overall changes in gloss happened in the first week, being relatively stable from the second to the eighth week of the CSTC (Figure 3d). Muñoz, et al.^20^ (2004) compared the efficacy of a low-abrasive calcium, phosphate, and sodium bicarbonate-based dentifrice with a high-abrasive silica-containing dentifrice *in situ* and showed that, after three months, the former improved the roughness and the gloss of enamel surfaces. Considering that gloss is result of the interaction between the light and the morphology of a surface, it seems safe to claim that the decrease in gloss in our study was due to the increase in the light scattering on the rougher enamel surfaces produced by the CSTC.

Although the results of our study add new and interesting aspects regarding the action of whitening toothpastes on enamel submitted to a cigarette smoking-toothbrushing-cycling, it should be kept in mind that it has several limitations. The use of bovine teeth, only two whitening toothpastes and one type of cigarette, and the short time of evaluation (eight weeks) are among the limitations. These and other aspects should be addressed in future investigations.

## Conclusions

Within the limitations of our study, we can conclude that the three toothpastes increased the surface roughness and were not capable of maintaining the optical stability (color, translucency and gloss) of enamel over a period of eight weeks submission to a cigarette smoking-toothbrushing-cycling. These results suggest that the therapy of using whitening toothpastes could be not totally efficient when used by heavy smokers.
